# The Macrophage Galactose-Type C-Type Lectin (MGL) Modulates Regulatory T Cell Functions

**DOI:** 10.1371/journal.pone.0132617

**Published:** 2015-07-06

**Authors:** Ilaria Grazia Zizzari, Paola Martufi, Federico Battisti, Hassan Rahimi, Salvatore Caponnetto, Filippo Bellati, Marianna Nuti, Aurelia Rughetti, Chiara Napoletano

**Affiliations:** 1 Department of Experimental Medicine, “Sapienza” University, Rome, Italy; 2 Department of Obstetrics and Gynecology, “Sapienza” University, Rome, Italy; Jackson Laboratory, UNITED STATES

## Abstract

Regulatory T cells (Tregs) are physiologically designed to prevent autoimmune disease and maintain self-tolerance. In tumour microenvironments, their presence is related to a poor prognosis, and they influence the therapeutic outcome due to their capacity to suppress the immune response by cell-cell contact and to release immunosuppressive cytokines. In this study, we demonstrate that Treg immunosuppressive activity can be modulated by the cross-linking between the CD45RA expressed by Tregs and the C-type lectin MGL. This specific interaction strongly decreases the im-munosuppressive activity of Tregs, restoring the proliferative capacity of co-cultured T lymphocytes. This effect can be attributed to changes in CD45RA and TCR signalling through the inhibition of Lck and inactivation of Zap-70, an increase in the *Foxp3* methylation status and, ultimately, the reduced production of suppressive cytokines. These results indicate a role of MGL as an immunomodulator within the tumour microenvironment interfering with Treg functions, suggesting its possible use in the design of anticancer vaccines.

## Introduction

Regulatory T cells (Tregs) (CD4^+^CD25^+^FOXP3^+^) play an essential role in the control of the immune response. They are crucial for maintaining peripheral tolerance and protection against autoimmunity, but they can also modulate immunity to infections and tumours [1]. In cancer, Tregs represent one of the main cellular subsets of the regulative network that are found in tumour microenvironments and are shown to be responsible for the negative regulation that occurs during antitumor immune responses [2]. This population exerts its regulatory activity through cell-cell contact and by producing suppressive factors such as interleukin (IL)-10 and Tumour Growth Factor (TGF)-ß. Several reports have shown that their presence in tumours is strongly related to the stage of disease and influences the outcome of the disease and therapy [3]. So far, several Treg subpopulations have been described, according to the co-expression of FOXP3 and CD45RA: CD45RA^+^FOXP3^low^ cells are resting Tregs (rTregs), CD45RA^−^FOXP3^high^ are activated Tregs (aTregs) and IL-17-secreting CD45RA^−^FOXP3^low^ are non-suppressive T cells (non-Tregs) [4]. CD45RA is one of the isoforms of CD45, a receptor-like protein tyrosine phosphatase expressed by all nucleated hemopoietic cells. CD45 represents the predominant transmembrane tyrosine phosphatase in lymphocytes and is required for the efficient induction of T cell receptor (TCR) signalling and activation [5]. In fact, CD45 dictates the switching of phosphorylation between the negative (Y505) and positive (Y394) regulatory Y residues of lymphocyte-specific protein tyrosine kinase (Lck kinase). Lck is inactive when Y505 is phosphorylated, while phosphorylation in Y394 triggers TCR activation and T cell proliferation through the Zeta-chain-associated protein kinase 70 (Zap-70) activation [6]. Among Treg subpopulations, the CD45RA^+^subset, expanded *in vitro*, gives rise to homogeneous Treg cell lines able to maintain FOXP3 expression and exhibit superior suppressive capacity [7,8]. The regulation of CD45RA activity in human Treg cells is poorly studied. CD45 expressed by effector T cells has been shown to recognise the macrophage galactose-type C-type lectin (MGL) through its *N*-acetylgalactosamine epitope (GalNAc), resulting in the negative regulation of T cells in terms of their proliferation and death [9]. MGL is a C-type lectin receptor expressed by activated macrophages and dendritic cells (DCs), which binds and internalises α- or ß-GalNAc (or Tn) residues of *N*- and *O*-glycans carried by self and non-self glycoproteins and/or glycosphingolipids [10]. Recently, we have shown the high plasticity of MGL and its capacity to modulate the immune response. MGL expressed by DCs mediates the binding and internalisation of tumour-associated antigen-carrying GalNAc residues, resulting in antigen cross-processing [11] and the induction of phenotypic and functional dendritic cell (DC) maturation [12]. In this work, we were challenged to verify whether the Treg subpopulation could also be affected by MGL engagement. The results demonstrate that CD45RA-MGL cross-linking decreases immunosuppressive activity of Tregs by changing CD45RA and TCR signalling and increasing *Foxp3* methylation accompanied by a reduced production of suppressive cytokines.

## Materials and Methods

### Treg isolation

Treg cells were purified from the buffy coat of healthy donors that were obtained from the Department of Haematology Sapienza University of Rome after informed consent, employing the human CD4+ CD127low CD25+ Regulatory T Cells kit (Stemcell, Canada) according to manufacturer's instruction. Briefly, CD4+ CD127low T cells were first pre-enriched from the buffy coat, and then T cells expressing high levels of CD25 were positively selected using EasySep Human CD25 Positive Selection Kit (Stemcell). Blood donor selection and collection was performed according to italian blood transfusion policy, regarding the signing of informed consent in which the donor agrees that his/her blood donation discard product can be used also for experimental research. Blood donations were anonymized.

### Flow cytometry

Cell phenotype staining was performed using the following panel of mouse monoclonal antibodies (MoAbs) anti-CD4-FITC, anti-CD25-PE, anti-CD45RA-PeCy5 and anti-FOXP3-Alexa647. For analysis of FOXP3, cells were first permeabilised with Human FOXP3 Buffer and then incubated with anti-FOXP3-Alexa 647. MoAbs anti-IgG_1_-FITC, anti-IgG_1_-PE, anti-IgG_1_-PeCy5 and anti-IgG_1_-Alexa647 were employed as isotype controls. All the MoAbs and reagents were from Becton Dickinson (San Diego, CA, USA). Treg cells were incubated with MoAbs for 30 minutes (min) at room temperature (RT). After washing, at least 10^4^ events were evaluated using a FACSCanto flow cytometer running FACSDiva data acquisition and analysis software (Becton Dickinson).

### Binding of rhMGL to Tregs and competition assay

The human recombinant MGL protein (rhMGL-Fc) was synthesised by GenScript USA Inc (Piscataway, NJ, USA). It contains the extracellular portion of the human MGL (MGL_396-476_) linked to the human Fc of IgG_1_. For binding, Tregs were incubated for 30 min on ice with rhMGL-Fc (10 μg/ml) in a solution of 20 mM Tris-HCL, pH 7.4, 150 mM NaCl, 2 mM MgCl_2_, 1 mM CaCl_2_ and 0.5% BSA (Sigma Chemical Company, St. Louis, MO, USA). Cells were extensively washed before any assay. To verify the binding of rhMGL to Treg, cells were stained with an anti-human IgG_1_ Fc-FITC (Jackson ImmunoReasearch Laboratories, West Grove, PA, USA). The binding was analysed by FACSCanto flow cytometer running FACSDiva data acquisition and analysis software. (Becton Dickinson). To evaluate the specificity of MGL binding, rhMGL-Fc was pre-incubated for 30 min at RT with GalNAc (20 μg/ml) and then added to Treg cells for 30 min on ice. Tregs alone were used as negative control.

### Immunoprecipitation and Western blot analysis

Immunoprecipitation was performed using the Immunoprecipitation Kit-–Dynabeads Protein G (Invitrogen, Oslo, Norway) according to manufacturer's instruction. Briefly, 50 μL of Dynabeads-protein G was added to 10 μg of rhMGL-Fc in 200 μL of Ab Binding & Washing Buffer for 20 min at RT. rhMGL-Fc coated beads were then washed and added to the cellular lysate of Tregs for 16 h at 4°C. The receptor coated to the beads was magnetically isolated and eluted using 20 μL of Elution Buffer. The concentration of eluted samples was evaluated using the Bradford assay (BioRad, München, Germany).

Immunoprecipitated samples were subjected to 3–8% or 4–12% sodium dodecyl sulphate-polyacrylamide gel electrophoresis (SDS-PAGE), and the resolved proteins were transferred electrophoretically to a nitrocellulose transfer membrane (Schleicher and Schuell, Dassel, Germany). The unspecific sites were blocked with phosphate-buffered saline (PBS) + 5% BSA (Sigma) overnight at 4°C. Nitrocellulose membrane was then incubated with anti-CD45RA antibody [MEM-56] (1:100) (Abcam) and with rhMGL-Fc (2,5 μg/mL) overnight at 4°C followed by peroxidase-conjugated goat anti-mouse IgG (H + L) and goat anti-human IgG, Fc fragment antibodies (1:20,000) (Jackson ImmunoResearch Laboratories, Suffolk, UK), respectively for 1 h at RT.

For the signal transduction experiments, Tregs were stimulated with rhMGL-Fc as described above. Cells were washed and incubated at 37°C for 20 min. Tregs were then lysed using the NP-40 solution (Biocompare) in the presence of phenylmethylsulfonyl fluoride (1 mM, PMES) (Sigma) and protease inhibitors (1X) (Sigma). Proteins obtained were resolved using 4–12% SDS-PAGE and transferred to nitrocellulose. After blocking, membranes were incubated with rabbit anti-pLck (Tyr 505) (Cell Signaling Technologies, Boston, MA, USA) (1:1000), rabbit anti-pLck (Tyr 394) (Santa Cruz, CA, USA) (1:1000), rabbit anti-Zap-70 (Cell Signaling Technologies) (1:1000) and mouse anti-β-actin (Santa Cruz) (1:1000) antibodies, followed by peroxidase-conjugated goat anti-rabbit or anti-mouse IgG (H+L) (Jackson ImmunoResearch Laboratories) (1:20000). The protein bands were detected with Lite Ablot Extend (Euroclone, Lugano, Switzerland) following the manufacturer’s instructions. The density of protein bands was analyzed by Image J software and was calculated in terms of average intensity of bands of each proteins per average intensity of bands of β-actin.

### Immunosuppression assay

CD4^+^ CD25^-^ T and Treg cells stimulated with rhMGL-Fc, were cultured alone or co-cultured (Tregs:CD4^+^ CD25^-^ T cells, 1:5) with anti-CD3 (1 μg/ml, OKT3) (eBioscience) and anti-CD28 (1 μg/ml)(BioLegend) antibodies for 4 days at 37°C. CD4^+^ CD25^-^ T cells was pre-treated with CarboxyFluorescein Succinimidyl Ester (1 μM, CFSE) (Life Technologies) and cell proliferation was monitored through progressive halving of fluorescence using FACSCanto flow cytometer. All experiments were performed in triplicate wells. One hundred percent proliferation was defined as the proliferation of CD4^+^ CD25^-^ T cells alone.

### ELISpot assay

PBMCs derived from buffy coat of healthy donors were co-cultured with K562 cells, pulsed with HLAI-A2 restricted Flu peptide (GILGFVFTL) (10 μg/mL) (Proimmune, Oxford, UK) for 12 days in RPMI+10% FCS in presence of IL-2 (10 U/ml) and IL-15 (10ng/ml). Flu-enriched CD8^+^ T cells were isolated employing the human microbeads CD8 (Miltenyi Biotech) and co-cultured with autologous Tregs (+/- rhMGL-Fc) and/or mDCs pulsed with Flu peptide in the anti-IFN-γ precoated (1:200; Pharmingen San Diego, CA, USA) ELISpot plates (Millipore, Bedford, MA, USA) overnight at 4°C. Cytokine release was detected with biotinylated anti-IFN-γ antibody (Pharmingen; 1:250, 2 hrs at RT) revealed with streptavidin-alkaline phosphatase (Pharmingen) (1:1000, 100 mL/well, 1 h at RT) and chromogen substrate (5-bromo-4- chloro-3-indolylphosphate (BCIP)/nitroblue tetrazolium alkaline phosphatase substrate, Sigma) (50 μL/well). Spots were counted using the ImmunoSpot Image Analyzer (Aelvis).

### *Foxp3* methylation analysis

Tregs, before and after stimulation with rhMGL-Fc protein, were lysed using Genomic Lysis Buffer (Zymo Research, Irvine, CA, USA) as indicated by the manufacturer’s instructions. Extraction of genomic DNA and *Foxp3* methylation analysis was performed by EpigenDx, Inc (Hopkinton, MA, USA) (Assay ID: ADS783FS2, Human Foxp3, TSDR region).

### Intracellular staining

Tregs after CD45RA stimulation were cultured with anti-CD3 (1 μg/ml)(eBioscience) and anti-CD28 (5 μg/ml) (BioLegend, San Diego, CA, USA) in presence of Brefeldin A (10 μg/ml) (Sigma) for 48 h at 37°C. Cells were collected, fixed and permeabilised with 0,5% saponin solution (Sigma). Intracellular staining was carried out using anti-IL-10-PE (Becton Dickinson), anti-IL-4-PeCy5.5 (Becton Dickinson), anti-IFN-γ-FITC (Becton Dickinson) and anti-IL-17-APC (eBioscience) antibodies. Cells were acquired by FACSCanto flow cytometer and analysed by FACSDiva software.

### Cell death analysis

Tregs were seeded in 96-well plates at a density of 10^5^/well and were cultured for 48 h with or without rhMGL-Fc (10 μg/ml) in the presence of anti-CD3 antibody (2,5 μg/ml) (eBioscience, San Diego, CA, USA). Apoptotic cells were evaluated using FSC/SSC as well as annexin-V-FITC (Becton Dickinson)/propidium iodide-PE (Becton Dickinson) cell staining.

### RNA isolation and reverse transcriptase Real-Time PCR assays

RNA extraction was performed with ReliaPrep RNA Cell Miniprep System (Promega, Madison, WI, USA) according to the manufacturer’s protocol. RNA (1 μg) was then converted into single-strand cDNA using high-capacity cDNA reverse transcription kits (Applied Biosystems, Foster City, CA, USA). Real-time PCR was carried out in a final reaction volume of 20 μl containing 25 ng cDNA, 10 μl TaqMan Universal Master Mix II (Applied Biosystems), 1 μl of each TaqMan Gene Expression assay (Applied Biosystems) (GATA3 Hs00231122_m1, RORC Hs01076122_m1, TBX21 Hs00203436_m1, B2M Hs99999907_1, RLP0 Hs99999902_m1) and DEPC-treated H_2_O (BioLine, Taunton, MA, USA). After denaturation at 95°C for 10 min, the samples were subjected to 40 cycles at 95°C for 15 sec and at 60°C for 1 min. All samples were analysed in triplicate using StepOne Real-Time PCR System (Life Technologies, USA). Gene expression levels were normalised using the 2^-ΔΔCt^ method.

### Statistical Analysis

Student's *t-*test was used for statistical analysis. Results with a p-value < 0.05 were considered significant.

## Results

### MGL recognises CD45RA on human Treg cells and reduces their suppressive activity

**Fig 1:**
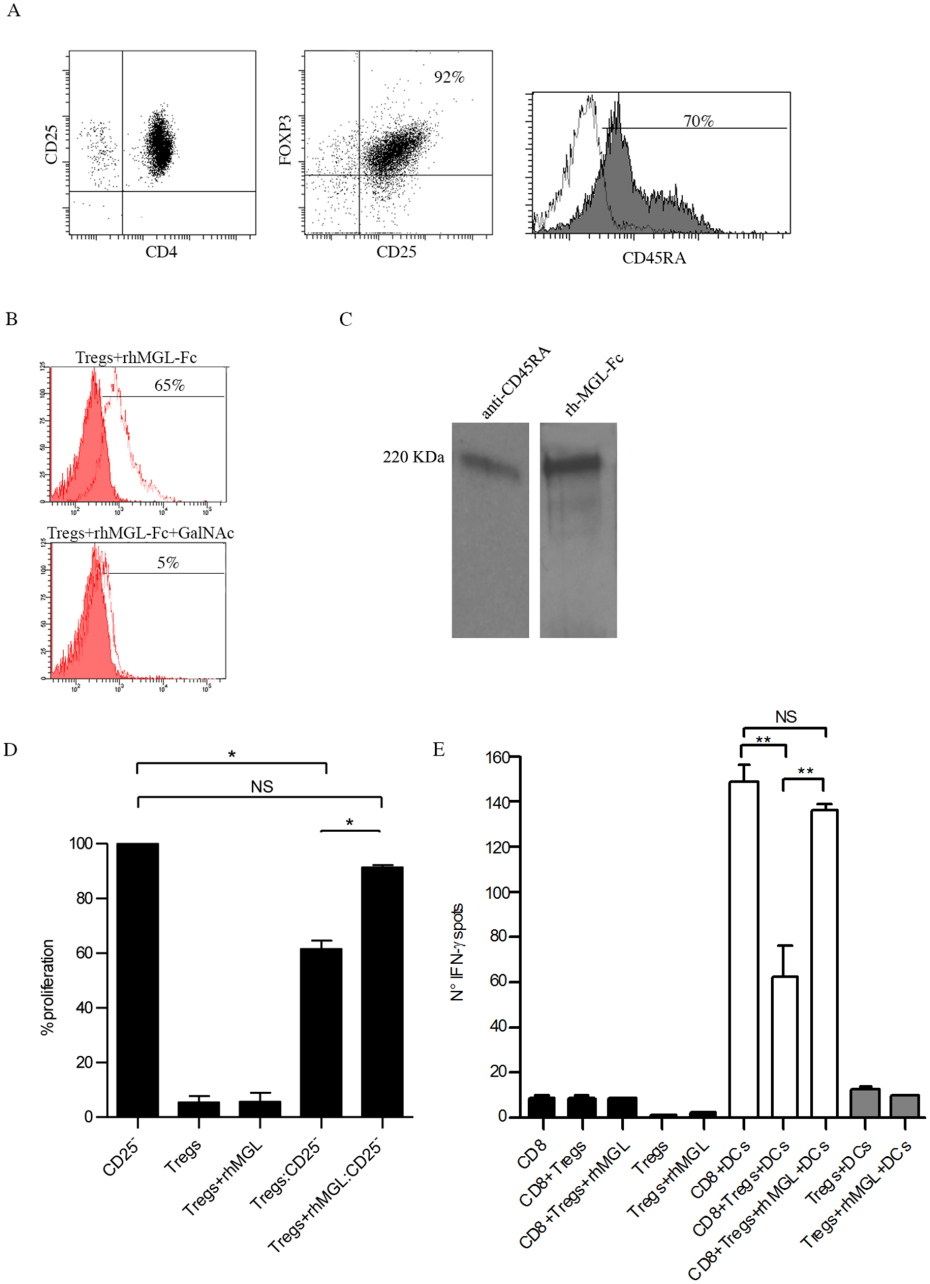
MGL recognises CD45RA on human Tregs and modulates their suppressive capacity. (A) Flow cytometry analysis of purified Treg stained with anti- CD4, CD25, FOXP3 and CD45RA antibodies. (B) Competition studies of rhMGL-Fc binding to CD45RA expressed by Tregs using GalNAc polymer. Filled histograms represent the untreated Tregs, while the open histograms show Tregs treated with rhMGL-Fc or rhMGL-Fc+GalNAc. (C) Western blot analysis of Treg lysate immunoprecipitated with rhMGL-Fc. Samples were run in 4–12% SDS-PAGE gel and were analysed with anti-CD45RA and rhMGL-Fc. These results are representative of one donor out of three. (D) Immunosuppression capacity of Tregs alone and Tregs treated with rhMGL-Fc after four days of co-culture with CD4^+^ CD25^-^ T cells (ratio 1:5, Treg:CD4^+^CD25^-^ T cells), in presence of anti-CD3 and anti-CD28. (E) IFNγ spots produced by CD8+ T cells (5×10^4^/well) stimulated with mDCs in presence or not of Tregs ± rhMGL. The results correspond with the mean of three independent experiments ± standard deviation (SD). * corresponds to *p*<0.05 and ** to p<0.01.

Tregs were isolated from PBMCs and characterised as shown in Fig 1A. After purification, we obtained 92% of Tregs (CD4^+^CD25^+^FOXP3^+^) of which 70% were positive for CD45RA.

MGL binding to CD45RA expressed by Treg cells was performed using a recombinant protein of MGL (rhMGL-Fc). rhMGL-Fc was made up of the extracellular domain of MGL fused to Fc domain of a human IgG_1_. We observed that MGL specifically bound Tregs on their surface (65%) and this interaction was abrogated when the GalNAc monosaccharide was incubated with rhMGL-Fc before binding (Fig 1B). Moreover, no Fc receptor (FcRs) expression was detected on Tregs, thus excluding the Fc portion of rhMGL-Fc as a participatory binding site (S1 Fig).

To understand if CD45RA was the unique MGL binder, we immunoprecipitated a Treg lysate with rhMGL-Fc. The immunoprecipitate was then analysed by western blot through probing of the blot with anti-CD45RA antibody and rhMGL-Fc (Fig 1C). When the membrane was incubated with the antibody, a line corresponding to CD45RA molecule (220 KDa) was observed. The same result was obtained using rhMGL-Fc to probe the blot. These data underline that CD45RA is the exclusive binder of MGL.

To study the effects of MGL binding on Treg activity, three independent immunosuppression assays were performed (Fig 1D). As expected, CD4^+^CD25^-^ T cells alone had maximum proliferative capacity (100%) after stimulation with anti-CD3 and anti-CD28 antibodies, while Tregs were anergic (8% proliferation) also after the stimulation with rhMGL-Fc (9,5%). Conversely, purified Tregs were able to inhibit CD4^+^CD25^-^ T cell proliferation, reducing their proliferative capacity from 100% to 60% (p<0.05). The proliferation of T cells was restored and increased from 60% to 90% (p<0.05) when rhMGL-Fc was added to the culture, suggesting that MGL-CD45RA interaction significantly modulates Treg function, reducing their immune suppression activity. Similar results were obtained in presence of antigen specific T cells (Fig 1E). Flu-enriched-CD8+ T cells, obtained from three healthy donors, were stimulated with autologous mDCs pulsed with HLAI-A2 restricted Flu peptide (GILGFVFTL) in presence or not of Treg±rhMGL-Fc. CD8+ T-cell activation was measured as IFN-γ production by ELISpot assay. Results indicated that mDCs induced a strong IFN-γ release when co-cultured with autologous CD8+ T-cells. The IFN-γ significantly decreased when Treg were added to the culture (p<0.01), while its production was restored when Treg were stimulated with rhMGL-Fc (p<0.01).

### *Foxp3* methylation is up regulated after MGL triggering

**Fig 2:**
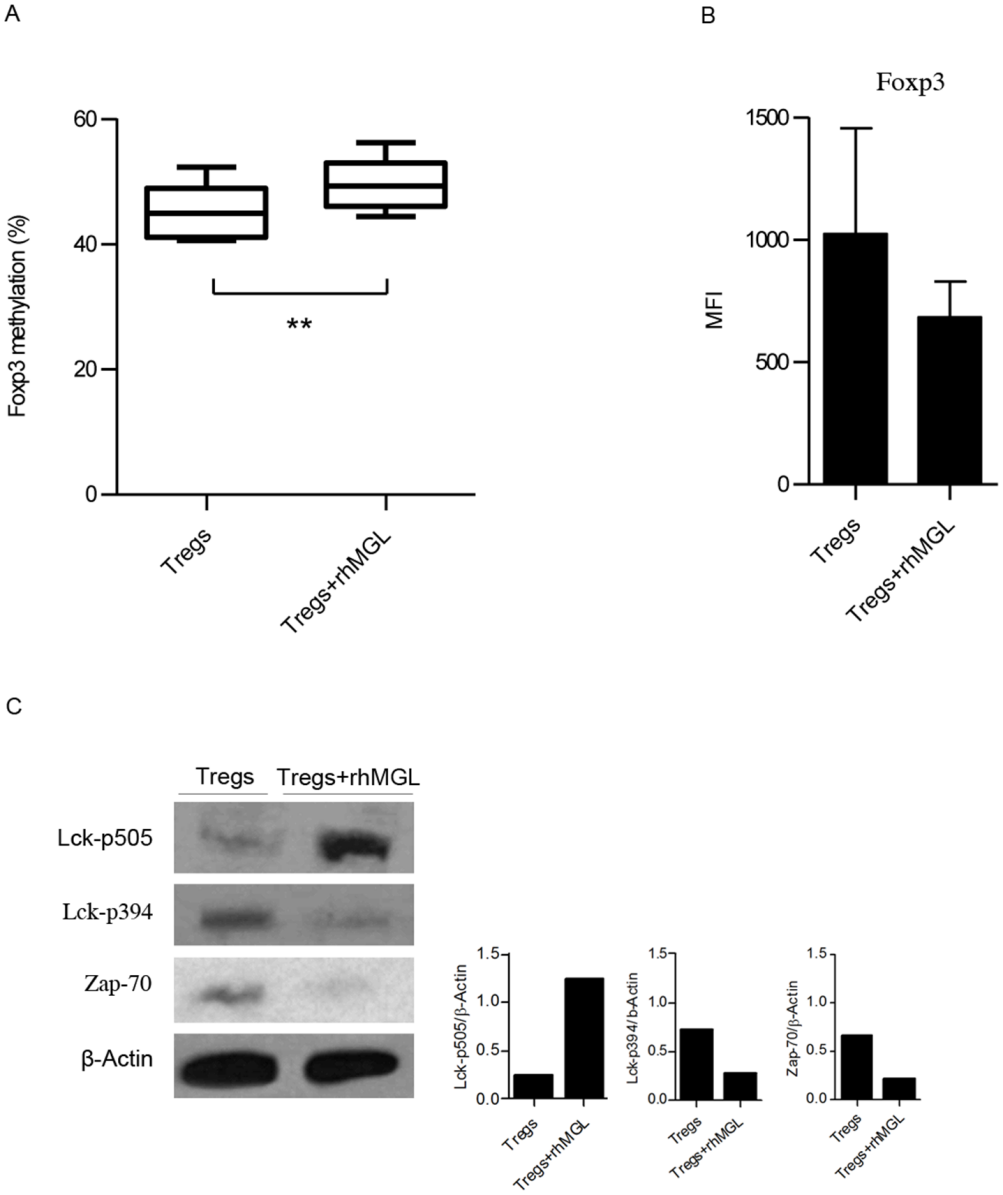
Binding of rhMGL-Fc to Tregs increases *Foxp3* methylation and downregulates TCR signalling. (A) Percentage of *Foxp3* methylation (*TSDR* region) of Treg cells alone or after treatment with rhMGL-Fc. The results correspond to the mean values obtained from three donors ± SD. (B) MFI values of FOXP3 expression of Tregs alone or after treatment with rhMGL-Fc stimulated with anti-CD3. The results correspond with the mean of three independent experiments ± SD. (C) Western blot analysis of Treg lysates with or without the treatment of rhMGL-Fc (left). Samples were analysed for pLck (Tyr 505), pLck (Tyr 394) and Zap-70. β-actin was used as a loading control. Proteins were resolved in 4–12% SDS-PAGE gel. Densitometric evaluation of the Lck-p505, Lck p-394, Zap-70 signals normalized to the levels of β-Actin (right). These results are representative of one donor out of three. ** Corresponds to *p*<0.01.

The development and immunosuppressive function of Tregs are regulated by the transcriptional factor FOXP3 [13]. The mechanism maintaining stable *Foxp3* expression in Tregs requires demethylation of the Treg specific demethylated region (*TSDR*) [14]. Therefore, increased methylation of this region reduces *Foxp3* transcription, impairing Treg function. To evaluate whether changes in *Foxp3* gene profiling could account for the decreased immunosuppressive activity of Tregs upon MGL-CD45RA interaction, we examined the methylation status of CpG sites in *TSDR*. Tregs alone and Tregs after rhMGL-Fc triggering were lysed for cDNA isolation and analysis. The results in Fig 2A show that the MGL binding on Tregs induced a significant increase of *Foxp3* methylation (p<0.01). In fact, in unstimulated Tregs, *Foxp3* methylation was 42%, while after MGL-CD45RA interaction, *Foxp3* methylation became significantly higher (50%; p<0.01).

Concurrently, Treg phenotype was monitored comparing Tregs treated with rhMGL-Fc to untreated Tregs. Tregs isolated from five healthy donors were characterised for the expression of CD4, CD25, CD45RA and FOXP3. In accordance with the data of *Foxp3* methylation, results show that MGL-CD45RA interaction also induced a reduction of FOXP3 expression (Fig 2B), although this modification was not statistically significant. CD4, CD25 and CD45RA were not modulated (data not shown).

### rhMGL-Fc-CD45RA interaction downregulates CD45 phosphatase activity

CD45 affects cellular responses by controlling the threshold of sensitivity to external stimuli. Therefore, to investigate whether MGL binding triggers changes in CD45RA intracellular signalling, we analysed the phosphatase activity of this receptor. Tregs alone and Tregs stimulated with rhMGL-Fc were lysed and probed with anti-pLck and anti-Zap-70 antibodies by western blot (Fig 2C). When CD45 dephosphorylates the negative regulatory tyrosine residue (Y505) on the protein kinase Lck, this kinase is activated thus triggering the TCR signalling. Reduced CD45 phosphatase activity after MGL-CD45RA interaction led to enhanced phosphorylation of Y505, thus decreasing Lck activity. Our results indicated that the binding of MGL to CD45RA induced a decrease in CD45 phosphatase activity with changes in Lck phosphorylation. After MGL-CD45RA interaction, Tregs exhibited strong phosphorylation of the Lck Y505 negative regulator, much higher than Tregs alone. Conversely, Tregs alone had Lck phosphorylated on Y394, the positive regulatory tyrosine residue. Moreover, because the activation of Lck challenges the induction of TCR signalling, we analysed the activation of Zap-70. When Treg cells were treated with rhMGL-Fc, Zap-70 resulted very weak. Instead, Tregs alone showed a strong activation of Zap-70 (Fig 2C). These data suggested that MGL-CD45 interaction modulates CD45 activity, switching the phosphorylation of Lck on negative residue Y505 and negatively influencing TCR signalling.

### MGL binding reduces immunosuppressive cytokines production but does not significantly affect Treg cell death

**Fig 3:**
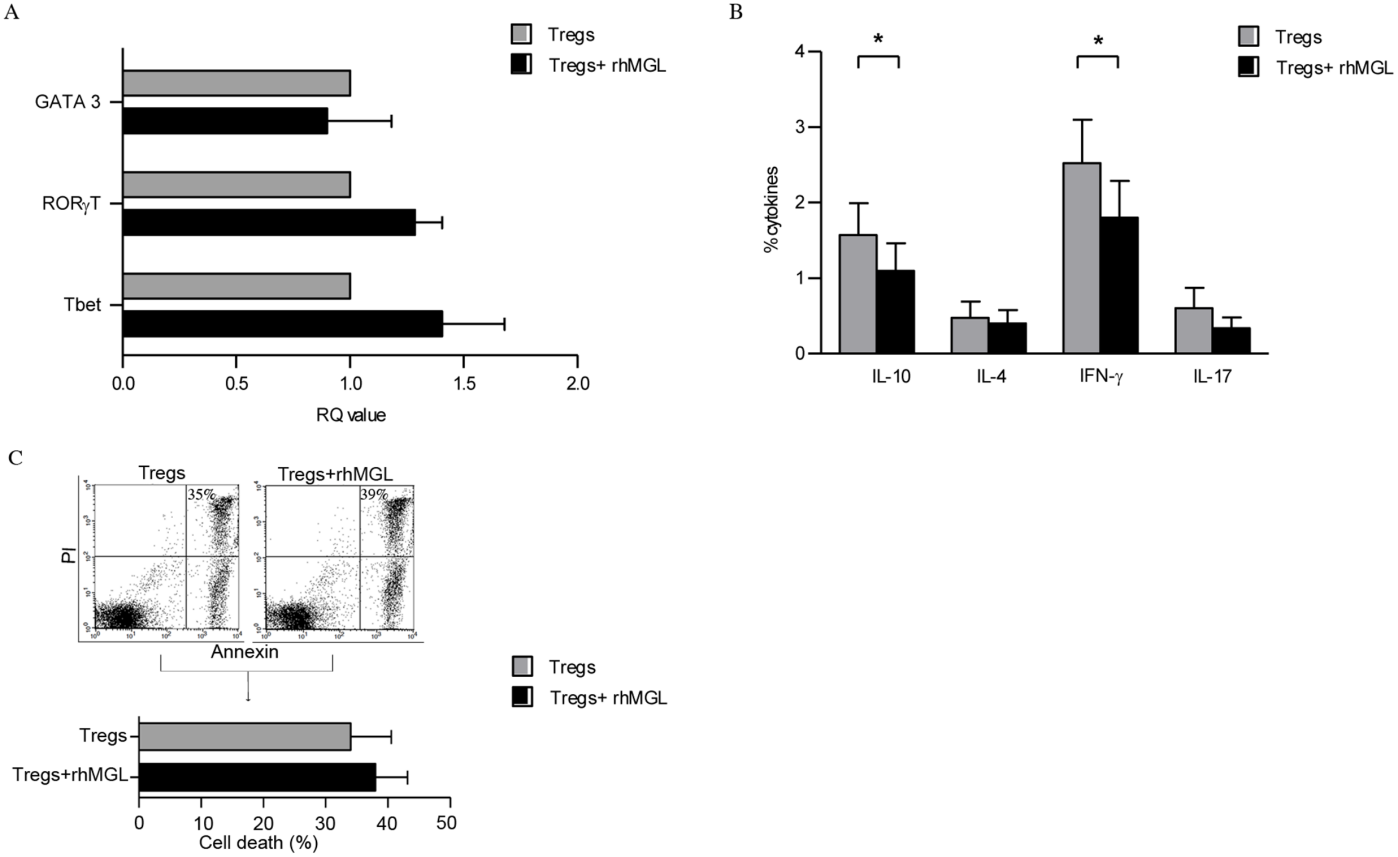
MGL-CD45RA interaction reduces immunosuppressive cytokine production. (A) Real-time PCR analysis of T-bet, RORγT and GATA3 genes in Tregs alone (grey columns) or after treatment with rhMGL-Fc (black columns). Relative gene expression was normalized with B2M and RLP0 gene expression levels. The results correspond to the mean value ± SD of three donors. (B) Intracellular staining of IL-10, IL-4, IFN-γ and IL-17 in Tregs alone (gray columns) or after treatment with rhMGL-Fc (black columns). The results correspond to the mean obtained from ten independent experiments ± SD. (C) Flow cytometry analysis of Tregs cultured with or without rhMGL-Fc and soluble anti-CD3. Quantification of cell death was performed using annexin-V-FITC and propidium iodide-PE. The results correspond to the mean value ± SD of three donors. * Corresponds to p<0.05.

We also investigated whether the reduced Treg suppression observed might be attributed to a conversion toward another specific phenotype (Th1, Th2, and Th17). cDNA isolated by Tregs alone and Tregs treated with rhMGL-Fc were analysed by RT-PCR for the expression of T-bet, GATA-3 and RORγt, which are the transcription factors responsible for Th1, Th2 and Th17 cell commitment, respectively. We observed no significant differences between Tregs alone and Tregs after CD45RA stimulation, suggesting that MGL triggering did not favour the switching of Treg cells toward a specific Th lineage (Fig 3A).

To confirm these data the production of IL-10, IL-4, IFN-γ and IL-17 by Treg cells was analysed. Tregs treated with rhMGL-Fc significantly reduced the secretion of IL-10 and IFN-γ (p<0.05; Fig 3B), while no difference in IL-4 and IL-17 production was observed.

Finally, we examined whether MGL-CD45RA interaction induced apoptosis of Treg cells. After 48 h of stimulation with anti-CD3 antibody in the presence of rhMGL-Fc, Tregs were stained with Annexin V and PI. We observed a trend showing an increase in cell death after triggering with rhMGL-Fc (-MGL vs. +MGL, 35% vs. 39%), although this result was not statistically significant (Fig 3C).

## Discussion

Treg-mediated immunosuppression is one of the crucial tumour immune-evasion mechanisms and represents the main obstacle of successful tumour immunotherapy. Tregs are physiologically designed to prevent autoimmune diseases by maintaining self-tolerance. These cells are able to inhibit the anti-tumour function of specific T cells; in fact their presence in tumour is directly related to poor prognosis [15].

Due to their important role involved in tumour progression, several strategies are under investigation to either manipulate the immune system to block the activities of suppressor cells or antagonise molecules or receptors involved in this process. These approaches are aimed to revert immunosuppression alone or in combination with molecules that target negative/positive T receptors, cancer vaccines or traditional chemotherapy [16, 17].

In this work, we demonstrate, for the first time, the role of the C-type lectin MGL in the modulation of Treg functions via CD45RA. In our previous studies, we have shown the important role of this receptor in the tumour-host dynamic interactions [11, 12]. We have proposed MGL as an optimal candidate receptor for DC-based immunotherapy due to the high plasticity and the capacity to modulate the immune response combined with the ability to endocytose and process GalNAc-carrying antigens. In this study, we show that MGL interacts with Tregs and regulates their functions through the binding to the CD45RA receptor. MGL specifically binds Tregs, shown in competition studies with the GalNAc where binding is abolished, and selectively recognises CD45RA on Treg cell surfaces, as shown by immunoprecipitation studies. In fact, CD45RA is the only molecule immunoprecipitated (220 KDa) by rhMGL, as shown by probing with anti-CD45RA antibody and with rhMGL. Previous studies have described that MGL, through the interaction of CD45, negatively regulates the activity of effector T cells (CD45RA^+^CD45RO^-^), decreasing T cell proliferation and increasing T cell death [9]. It is well known that CD45 represents a key molecule fine-tuning T cell-function. The engagement of CD45 through MoAbs has been successfully employed to modulate T cell responses inducing tolerance *in vivo* and *in vitro* transplant models [18, 19].

T cells can express different isoforms of CD45 such as CD45RA and RO, according to the activation status of the cells. In Treg subpopulations, CD45RA expression is associated with the unique Treg subset able to maintain, *in vitro*, the suppressive activity given by a stable expression of FOXP3 [7, 8]. Employing a recombinant MGL molecule and purified Treg cells expressing CD45RA, we have observed that the binding between MGL and CD45RA expressed by CD45RA^+^FOXP3^+^ Tregs reduces the suppressive activity of this cell subset restoring the proliferative capacity of T cells.

The crucial role of CD45 protein tyrosine phosphatase promoting T cell activation and development [20] is primarily attributed to the positive regulatory role of CD45 in fostering the activity of Lck, a Src family kinase. In fact, Lck helps initiate TCR-mediated signal transduction pathways by inducing tyrosine phosphorylation of CD3/ζ ITAM residues as well as association and activation of the Zap-70 tyrosine kinase [21]. The CD45-MGL interaction also leads to Lck inactivation within the Treg subpopulation, as described for the effector counterpart [9]. Moreover, we show that this phospho-signalling leads to inactivation of Zap-70, resulting in the TCR signalling block.

The decrease of Treg suppressive activity did not correlate with cell death. Although we detected CD45-dependent apoptosis in human Treg after MGL binding, this was not significant. This result is in agreement with what was described in Treg mouse models in which apoptosis did not correlate with the abrogation of Treg suppression activity upon cross-linking of CD45RO with a specific MoAb [22].

It is well known that stable expression of FOXP3 is an essential prerequisite for the maintenance of suppressive properties of Tregs. Stable *Foxp3* expression is associated with selective demethylation of an evolutionarily conserved element within the *Foxp3* locus named *TSDR* [14] although a direct correlation between the degree of *TSDR* methylation and protein level of FOXP3 was not observed [23]. We found that MGL-CD45RA cross-linking significantly increased the methylation status of *TSDR*, thus reducing the immunosuppressive activity of Tregs, while only a trend toward the decrease of FOXP3 protein was observed.

Several studies provide evidence that Treg differentiation is an irreversible process. However, more recently data indicates that Treg cells retain developmental plasticity, namely the capacity to be reprogrammed into various Th cell subsets depending on the extrinsic stimuli used [24, 25]. Treg stimulated with MGL lose part of their suppressive activity, but CD45RA-MGL interaction was not sufficient to favour the switching of Tregs toward a specific Th lineage. We observed no increase of the specific transcription factors that characterised the Th1, Th2 and Th17 lineage (Tbet, GATA3 and RORγt, respectively) and no increase in the production of IL-4 and IL-17. Moreover, we detected a significant decrease in IL-10 and IFN-γ. Tregs are able to produce several type of cytokines. IL-10 represents the cytokine required for their immunosuppressive activity [26]. Additionally, CD4^+^CD127^low^CD25^+^ Tregs expressing high levels of FOXP3 with a high suppressive capacity produced IFN-γ. The decrease in IL-10 and IFN-γ could account for the reduction of Treg suppressive activity observed in our *in vitro* system.

In this study, we describe for the first time a novel mechanism whereby Treg functionality can be overcome, depending on the interaction between CD45RA and the C-type lectin MGL. This interaction is able to modify the epigenetic signature of *Foxp3* and signalling pathways, thus reducing the immunosuppressive potential of Treg CD45RA^+^FOXP3^+^, i.e., a lower production of immunosuppressive cytokines and restoration of effector T cell proliferation.

We are currently investigating the MGL-CD45RA interaction between DCs and Tregs to better understand the relevance of this interaction in an *in vitro* cell system.

The reported results herein strengthen the role of MGL as immunomodulator of the tumour microenvironment able to interfere with Treg functions in addition to mediate glycoantigen up-take and DC activation. Therefore, these results strongly suggest its employment in the design of anticancer vaccines.

## Supporting Information

S1 Fig Tregs do not express Fc receptors. CD16, CD32 and CD64 expression was analyzed by Flow cytometry. The results are representative of 1 donor out of 3
(TIF)
