# Supplementary figures and images for: The Macrophage Galactose-Type C-Type Lectin (MGL) Modulates Regulatory T Cell Functions
Source: PLoS One. 2015 Jul 6;10(7):e0132617. doi: 10.1371/journal.pone.0132617 (PMC4493043; doi:10.1371/journal.pone.0132617)

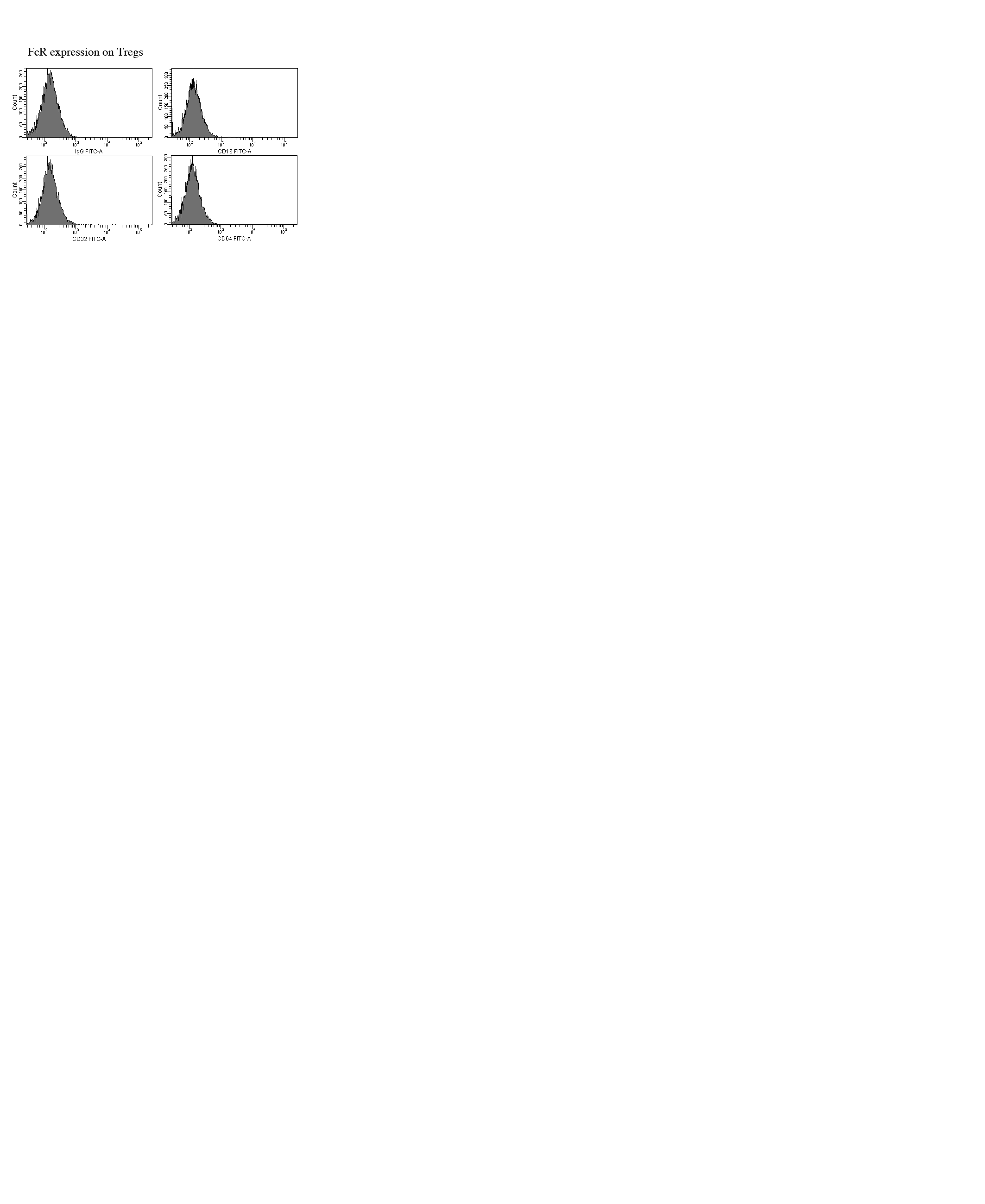

Supplement: S1 Fig — CD16, CD32 and CD64 expression was analyzed by Flow cytometry. The results are representative of 1 donor out of 3 (TIF) [file pone.0132617.s001.tif]
